# Understanding barriers and facilitators to doxycycline post-exposure prophylaxis adherence among young women in western kenya: a qualitative study

**DOI:** 10.1186/s12879-025-11209-6

**Published:** 2025-07-01

**Authors:** Benn Kwach, Zachary Kwena, Lauren R. Violette, Bernard Rono, Josephine B. Odoyo, Kevin Oware, Elizabeth A. Bukusi, Jared M. Baeten, Lucy Mkandawire-Valhmu, Jenell Stewart, dPEP Kenya Study Team, Alfred Odira, Perez Ochwal, Lydia Adiema, Marion Hewa, Elizabeth Koyo Akumu, Linda Aswani, Lawrence Juma, Violet Kwach, Felix Mogaka, Vincent Momanyi, Alfred Obiero, Loice Okumu, Victor Omollo, Christine Otieno, Greshon Rota, Jacqueline M. Amira, Justice Quame-Amaglo, Ruanne Barnabas, Jennifer Baugh, Jade Boyer, Connie Celum, Kristin Cicciarella, Deborah Donnell, Daphne Hamilton, Harald Haugen, Rachel E. Johnson, Toni M. Maddox, R. Scott McClelland, Susan A. Morrison, Colin S. Pappajohn, Elena Rechkina, Caitlin Scoville, Tina Sesay, Olusegun O. Soge, Kathy Thomas, Jane Simoni, Vianey Vazquez Venegas

**Affiliations:** 1 Centre for Microbiology Research, Kenya Medical Research Institute, P. O. Box 614-40100, Kisumu, Kenya; 2 Department of Population Medicine, Harvard Pilgrim Health Care Institute, Boston, USA; 3 Departments of Global Health, University of Washington, Seattle, USA; 4 Departments of Ob/Gyn, University of Washington, Seattle, USA; 5 Departments of Infectious Diseases, University of Washington, Seattle, USA; 6 School of Nursing, University of Minnesota, Minneapolis, USA; 7 Department of Medicine, University of Minnesota, Minneapolis, USA; 8 Division of Infectious Diseases, Hennepin Healthcare, Minneapolis, USA

**Keywords:** Adherence, Barriers, Facilitators, DoxyPEP, STIs, HIV, Pre-Exposure Prophylaxis, Young Women, Qualitative Study, Kenya

## Abstract

Sexually transmitted infection (STI) rates are high globally, particularly in regions like sub-Saharan Africa, where there is also a disproportionate burden of HIV*.* Doxycycline post-exposure prophylaxis (doxyPEP) is a novel strategy intended to prevent bacterial STIs following potential exposure. The dPEP Kenya Study, the first trial of doxyPEP for STI prevention among cisgender women, found that doxyPEP did not reduce STIs in the setting of low use of doxyPEP by objective drug concentrations. To assess barriers and facilitators to doxyPEP adherence, we explored participants' experiences during the dPEP Kenya Study. We conducted serial in-depth interviews (n = 40) and 4 focus group discussions (n = 29) of participants randomized to take doxyPEP. Transcripts were analyzed using an inductive content analysis approach. Side effects, such as nausea from taking doxyPEP on an empty stomach, dosage interpretation challenges, pill burden, and concerns about stigma and partner reactions hindered adherence. Support from partners, family, and peers, familiarity with doxycycline, and the use of discreet pill carriers facilitated doxyPEP use. Decreasing dosage frequency, promoting the use of discreet pill carriers, and addressing stigma through community-driven communication strategies may improve future doxyPEP uptake and adherence. Clinical trial number. This trial was registered in the ClinicalTrials.gov under registration number, NCT04050540 on 06th August 2019.

**Supplementary Information:**

The online version contains supplementary material available at 10.1186/s12879-025-11209-6.

## Background

Sexually transmitted infections (STIs) continue to pose a significant public health challenge globally, especially among adults in sub-Saharan Africa. Around the world, STIs are increasing among people engaged in HIV pre-exposure prophylaxis (PrEP) care, highlighting the syndemic of HIV and curable STIs. In Kenya, PrEP scale-up began in 2017, and young women are a priority group for PrEP. PrEP Trials in Kenya revealed a high incidence of STIs among female participants at rates comparable to those described in men who have sex with men (MSM) in the USA and Europe [1].

In sub-Saharan Africa, young women are disproportionately affected by a high incidence of curable STIs, which exacerbates the impact of both HIV and other infections [2]. Cisgender women aged 15–24 years account for the majority of new STI cases, which can lead to complications such as infertility, ectopic pregnancy, and increased susceptibility to HIV infection [3].

Doxycycline post-exposure prophylaxis (doxyPEP) is a novel strategy of taking 200 mg of doxycycline within 72 h of possible exposure to prevent bacterial STI [4]. DoxyPEP is proven to reduce the incidence of chlamydia, syphilis, and gonorrhea among MSM and transgender women [5, 6]; however, the only trial of doxyPEP among cisgender women, the dPEP Kenya Study, did not demonstrate efficacy and participants were found to have low use of doxyPEP in the study [7].

The adoption of efficacious interventions that prevent HIV and STIs is essential to reducing their incidence globally; however, understanding factors that influence adherence to medications, like PrEP and doxyPEP, is equally important for assessing effectiveness [8]. Stigma surrounding sexual activity and the use of preventive care can manifest in various forms, including fear of judgement or discrimination, and can be a major barrier to accessing and adhering to these interventions [9–15]. Missed doses of preventive medications, like HIV PrEP, have been linked to intimate partner violence and trust deficits, which further complicate adherence for young women in relationships that often increase risk of HIV and STI acquisition [16–21].

While several studies have explored the acceptability of doxyPEP among key populations such as MSM, limited data exist on the barriers and facilitators of doxyPEP use among cisgender women [22]. The goal of this qualitative study is to explore the perspectives of women participating in the dPEP Kenya Study on barriers and facilitators of medication adherence to further contextualize null results of the trial. The insights gained are critical in guiding future efforts to doxyPEP scale-up as a preventive measure for bacterial STIs.

## Methods

### Study Design

We conducted a qualitative evaluation of participant experiences in the dPEP Kenya Study, an open-label randomized controlled trial of doxyPEP to prevent STIs among cisgender women [7, 23, 24]. Between June 2021 and August 2023, a subset of participants randomized to the doxyPEP intervention participated in serial in-depth interviews at three-time points during the study and focus group discussions following study completion.

### Study Setting and Participants

**Fig. 1:**
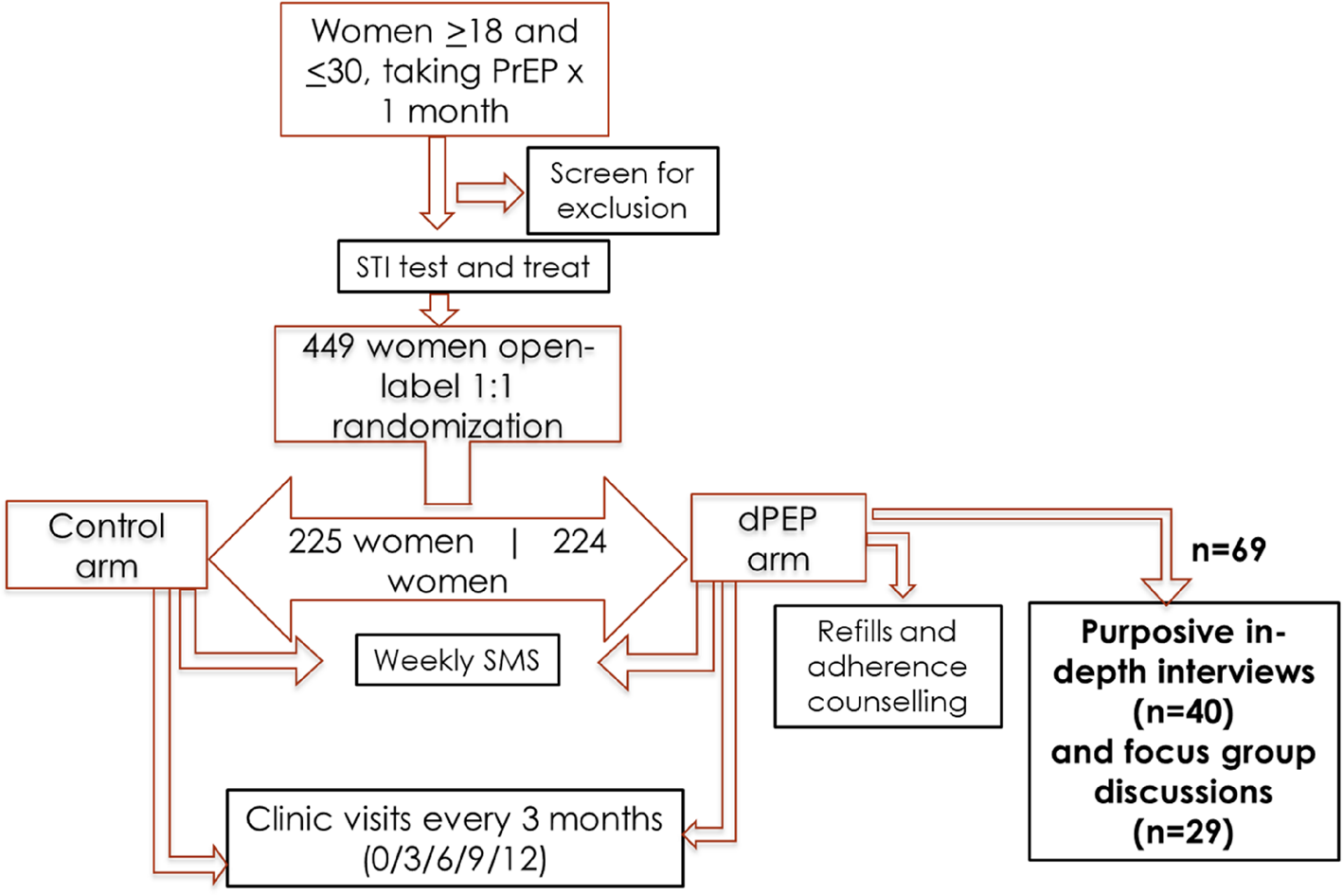
The dPEP Kenya Study design summary

The dPEP Kenya study enrolled 449 cisgender women aged 18 to 30 years, all using daily oral HIV pre-exposure prophylaxis (PrEP) in Kisumu, Kenya. Participants were followed for 12 months with quarterly STI testing, treatment, and adherence counselling with detailed description of trial design previously published [7, 23]. The qualitative component involved 69 purposively sampled participants from the 224 women in the intervention (doxyPEP) group. Participants were required to be sexually active and have self-reported doxyPEP use at least twice within a month to allow for a rich exploration of experiences with doxyPEP. Figure 1 illustrates the study design and flow. Participants who became pregnant were advised to discontinue doxyPEP during pregnancy.

### Data Collection

Surveys on demographics and sexual behavior were collected at the time of enrolment in the trial. A total of 120 semi-structured in-depth interviews (IDIs) were conducted with 40 participants, each completing three serial interviews: within one month of enrolment (to capture early experiences), at Month 6 (after gaining substantial experience with doxyPEP), and at Month 12 (to assess changes over time). The interviews enabled us to compare experiences by participant over time.

Additionally, four FGDs (n = 29) were conducted between June and August 2023 after study completion to further explore participants’ experiences with doxyPEP and their views on why the intervention did not demonstrate efficacy in preventing STIs.

Participants were purposively sampled from the broader trial population based on their self-reported adherence to doxyPEP, aiming to capture a range of adherence experiences. This diversity allowed us to explore varying perspectives and contextual factors influencing adherence. The 29 FGD participants were sampled independently from the 40 IDI participants, with no overlap between the two groups.

All interviews and discussions were conducted in the participants’ preferred language (either English, Swahili, or Dholuo), and were audio-recorded, transcribed, and translated into English when necessary. To ensure accuracy of translation of interviews done in Swahili or Dholuo, four independent consultants translated the entire interviews to English before another set of four consultants backtranslated from English into either Swahili or Dholuo per language used in the interviews. Participants provided informed consent and were reimbursed 500 Kenyan Shillings (about USD 3.86) for travel and to compensate them for their time and effort. The complete English-language versions of the interview guides are provided as Supplementary File 1 (IDI Guide) and Supplementary File 2 (FGD Guide).

### Data Analysis

Qualitative data were analyzed using an inductive content analysis approach [25] to identify key themes and patterns related to doxyPEP adherence. Transcripts from the serial interviews were used to iteratively develop a codebook, which guided the coding process. Six coders (LA, MH, AO, PO, BK, and ZK) independently coded two transcripts, after which discrepancies in code application were discussed and resolved, and the codebook was iteratively refined to enhance intercoder reliability. For the FGDs, a rapid analysis approach, [26], which involved summarizing and interpreting data using a structured template, was applied to identify key themes. The final transcripts were coded independently, and code reports were generated to facilitate content analysis. Data from both IDIs and FGDs were imported into Dedoose [27], and findings were organized into a framework analysis table, highlighting barriers and facilitators to event-driven doxyPEP use.

### Trustworthiness

The study’s trustworthiness was ensured through the principles of credibility, transferability, dependability, and confirmability [28]. Credibility was established through prolonged engagement, as the research team maintained sustained involvement with the community partner agencies even before the study commenced. Community Advisory Groups (CAGs) met twice a year to provide necessary input and feedback on the study. We also partnered with PrEP-implementing partners, community-based organizations (CBOs), and community health volunteers (CHVs) and provided them with regular updates on the study. This engagement allowed for an in-depth understanding of the context and helped build trust with participants and the community. On transferability, a thick description of the study themes is provided to enable readers to assess the relevance and applicability of the findings to other settings. Dependability and confirmability were maintained by creating an audit trail and documenting decisions and processes throughout the research. This ensures transparency and that the study findings are grounded in the data collected, while also providing a pathway for replication in similar contexts.

## Results

### Socio-demographic Characteristics

Participants (N = 69) were drawn from Kisumu County and the majority were aged between 18 and 25 years (38/69, 55.1%) and never married (50/69, 72.5%). Over half (43/69, 62.3%) were employed in the informal sector, and more than half (37/69, 53.6%) engaged in transactional sex in the prior 3 months, defined as having a sex partner who provided them with money or gifts, such as airtime, clothes, cell phones, food, or cosmetics. Table 1. These characteristics are broadly representative of the larger randomized doxycycline PEP group, with minor variations observed in education, income, and engagement in transactional sex.

**Table 1:** Socio-Demographic Characteristics

Characteristics		Qualitative participants, N = 69 n (%)	dPEP Kenya Doxycycline PEP group, *N* = 224 n (%)
**Age**	18–25	38 (55.1%)	157 (70.1%)
	26–30	31 (44.9%)	67 (29.9%)
**Marital Status**	Never married	50 (72.5%)	158 (70.5%)
	Married	11 (15.9%)	39 (17.4%)
	Separated/Divorced	8 (11.6%)	27 (12.1%)
**Living with a Partner**	Yes	13 (18.8%)	40 (17.9%)
	No	54 (78.3%)	177 (79.0%)
	No partner	2 (2.9%)	7 (3.1%)
**Education Level**	Primary school incomplete	3 (4.3%)	16 (7.2%)
	Primary school complete	10 (14.5%)	33 (14.7%)
	Secondary school incomplete	14 (20.3%)	54 (24.1%)
	Secondary school complete	27 (39.1%)	81 (36.2%)
	Post-secondary education	15 (21.7%)	40 (17.9%)
**Employment Status**	No personal income	22 (31.9%)	87 (38.8%)
	Informal sector employment (e.g., trader, selling goods)	43 (62.3%)	121 (54.0%)
	Other	4 (5.8%)	16 (7.1%)
**Engages in Transactional Sex**	Yes	37 (53.6%)	89 (39.7%)
	No	32 (46.4%)	135 (60.3%)

### Barriers to DoxyPEP Adherence

Several barriers to adherence were identified, with certain themes present throughout the study and others more prominent at specific stages. The main barriers to doxyPEP adherence identified in this study are the side effects, stigma and privacy, forgetfulness and routine disruptions, and partner influence and reactions.

#### Side Effects

Side effects were reported by 28 participants at Month 1, 15 participants at Month 6, and 25 participants during the exit interviews. It was a recurring theme across IDIs and FGDs. Side effects, such as nausea, fatigue, and vomiting were frequently reported as a major barrier to adherence. These side effects, primarily associated with DoxyPEP and not PrEP, were uncomfortable and sometimes severe, leading to non-adherence or doubts about continuing with the medication. Participants reported that side effects, related to gastric irritation, were most often prominent when doxycycline was taken on an empty stomach. As a result, they did not take the tablets regularly. This was confirmed by many participants who expressed that the adverse effects made it difficult to continue taking doxyPEP as prescribed. One participant stated, *“I didn’t take the full doxyPEP dose due to severe side effects. I had constant nausea and couldn't eat without vomiting. It was too much for me to handle”* (28 years old, divorced, primary school not completed- Month 1 IDI).

Another added, *“Taking doxyPEP sometimes makes me nauseous. If I have to work soon, I avoid taking it because it affects me right away”* (25 years old, never married, attended post-secondary school- FGD 2).

Managing multiple medications was overwhelming for some participants, particularly due to the pill burden of taking daily HIV PrEP alongside doxyPEP. This challenge was heightened when participants had to take additional medications for other illnesses. One participant explained, *“Taking so many pills at the same time is really tough, especially when I get sick with something like malaria. I already have doxyPEP and my daily PrEP, and then adding more just feels overwhelming. Sometimes I even forget a dose or skip because it’s just too much to handle”* (22 years old, never married, secondary school completed—Month 12 IDI). Another participant highlighted the challenge of taking PrEP and 200 mg of doxyPEP stating, *“It is a challenge because they are now many, they are now three!”* (20 years old, never married, secondary school completed—Month 6 IDI).

#### Stigma and Privacy Concerns

Stigma and privacy concerns were mentioned by 21 participants at Month 1, 27 at Month 6, and 15 during the exit interviews. Participants spoke about fears of being judged by others or had concerns about being perceived as engaging in risky sexual behavior, and this hindered adherence. Stigma, tied to perceptions of promiscuity and assumptions of STI infection due to the use of doxyPEP and concerns about privacy, were significant barriers for many participants. Stigma associated with STIs and misconceptions about doxyPEP led participants to hide their medication use or avoid disclosing it to others, including partners and family members. One participant expressed, *“When I am in a new environment, I start asking myself what people will think when they see me taking doxyPEP”* (25 years old, never married, attended post-secondary school- Month 6 IDI).

#### Forgetfulness and Routine Disruptions

Forgetfulness and routine disruptions was a theme mentioned by 8 participants at month 1, 16 at month 6, and 20 during the exit interviews. Forgetfulness, often exacerbated by changes in routine, such as travel or work commitments, was another common barrier to adherence. A participant shared, *“I went to work without it and forgot about the reminder. I was far from my phone at the time. The next day, the reminder went off again, and I remembered I hadn't taken it the previous day. However, I was far from my doxyPEP at that moment too since I forgot to carry them”* (30 years old, never married, completed secondary school- Month 12 IDI).

#### Partner Influence and Reactions

Partner influence and reactions was a theme noted by 13 participants at Month 1, 14 at Month 6, and a smaller group during the exit interviews. Partner reactions played a significant role in adherence. Negative reactions (such as questioning the necessity of DoxyPEP, fear of infidelity, as mentioned earlier, and concerns about reduced fertility resulting from DoxyPEP), scepticism, or opposition from partners to doxyPEP use, influenced participants' decisions and adherence behaviors. Some participants encountered resistance or disapproval from their sexual partners regarding doxyPEP use, which hindered their ability to adhere to the medication.

*“I stopped taking doxyPEP because my partner and I used to argue about it. When you’re with someone who is serious about settling down with you, seeing you take a lot of medication might make them think you're still involved in your past lifestyle (infidelity) or returning to old habits.”* (23 years old, never married, completed secondary school- FGD 2).

### Unique Barriers at Specific Time Points:

It is important to note that there were some barriers that were most prominent at specific time points. For example, pregnancy and health concerns peaked at month 6 as participants became more aware of potential risks, while access issues surfaced at month 12 due to reduced support and increased travel or relocation for work, school, or other personal reasons.

#### Pregnancy and Health Concerns

In month 6, 5 participants expressed concerns about the potential impact of doxyPEP on pregnancy and overall health. These worries influenced their decision to stop or reduce adherence during this time.

#### Access Issues

During the exit interviews, 5 participants reported logistical challenges in accessing doxyPEP, especially if they had relocated or experienced transportation difficulties. These access issues became more prominent as the study neared completion.

### Facilitators to doxyPEP Adherence

Several themes were identified from the data as facilitators to adherence, with some recurring across all time points and methods, while others were more prominent at specific stages of the study. The main facilitators to doxyPEP adherence identified in this study are the perceived effectiveness of doxyPEP in STI prevention, use of discreet pill carriers, social support and encouragement and routine integration.

#### Perceived Effectiveness of doxyPEP in STI Prevention

Perceived effectiveness of doxyPEP was consistently reported by 12 participants at Month 1, 15 participants at Month 6, and 30 participants during the exit interviews. The perception of protection against STIs reinforced adherence across both IDIs and FGDs.

A strong motivator for adherence was the belief in doxyPEP’s effectiveness in preventing STIs. Participants frequently expressed confidence that doxyPEP provided them with protection.

*“I don’t feel any difficulty in using doxyPEP because it protects me from STIs”* (22 years old, never married, completed secondary school- Month 1 IDI).

#### Use of Discreet Pill Carriers

The use of discreet pill carriers was mentioned by 10 participants at Month 1, 16 at Month 6, and 15 during the exit interviews. The ability to maintain privacy, by using pill carriers, without raising suspicion from others in their households or social circles, supported the continuous use of doxyPEP.

All participants randomized to the intervention arm were provided with pill carriers from empty lipstick containers. These discrete pill carriers were beneficial for their convenience and ability to disguise the medication, making it easier for participants to carry and use doxyPEP without drawing unwanted attention. Many participants found using pill carriers that resembled everyday items, such as small cosmetic containers, facilitated adherence by allowing them to take the medication discreetly.

*“Carrying the whole box of medicine when your purse is small is hectic, so inside the pill carrier you can put a few that you can use without anyone knowing what it is since it resembles a lipstick container”* (23 years old, never married, secondary school not completed- Month 6 IDI).

#### Social Support and Encouragement

Social support and encouragement was a theme mentioned by 9 participants at Month 1, 14 at Month 6, and 20 at Month 12. Supportive social environments, including friends, family, and partners who understood the importance of doxyPEP, played a significant role in promoting adherence by offering emotional encouragement, reassurance and approval. Some participants described how open conversations about doxyPEP use and ongoing support from trusted individuals helped them feel more confident and motivated to stay consistent with the medication. A participant shared, *“I told my friends, sisters, and parents that doxyPEP prevents STIs, so I don’t have any problem since they encouraged me to use it”* (20 years old, never married, completed secondary school- Month 12 IDI).

Participants reported that encouragement and emotional support made it easier to adhere to their regimen. Another participant shared how parental support influenced her decision, stating, *“She (mother) told me to take them so long as it doesn’t cause me harm—so this has made it easier for me to take them.”* (19 years old, never married, completed secondary school- Month 12 IDI).

#### Routine Integration

Routine integration was mentioned by 7 participants at Month 1, 14 at Month 6, and 25 during the exit interviews. Integrating doxyPEP into daily routines, such as associating it with mealtimes or aligning it with PrEP use, emerged as a practical strategy to ensure adherence. Participants who had a structured routine reported fewer instances of forgetting to take their pills.

### Unique Facilitators at Specific Time Points

#### Health Education and Provider Support

Some participants highlighted the importance of health education and support from healthcare providers as critical facilitators to their continued use of doxyPEP. This was noted by 8 participants, who reported that regular check-ins with providers reassured them and kept them engaged with the intervention.

*“The staff here influence me from the way they educate us to protect ourselves from STIs and HIV. My prescriber is friendly and always encourages me to keep taking PrEP and doxyPEP.”* (19 years old, never married, secondary school completed- Month 6 IDI.)

#### Fear Reduction

In Month 6, one participant shared that their adherence improved as initial fears—such as concerns about side effects or social judgment—diminished over time. This personal experience highlights how individual perceptions can shift with continued use.

*“My fear of getting infected with an STI has also reduced even if I was to have unprotected sex. That’s why I feel that doxyPEP is good. It’s quite easy for me because I do not feel any side effects when I take it these days, I just feel okay.”* (28 years old, divorced, primary school not completed- Month 6 IDI).

#### Positive Experiences and Benefits

Positive experiences and benefits were prominently mentioned by 7 participants in month 6 and reinforced their belief in the personal health benefits of doxyPEP. Participants who had positive experiences with doxyPEP, such as relief from symptoms or improved sexual health, found it easier to continue using the medication. One participant noted, *“I can say it has been helping me because previously after having sex I used to feel itchiness, so nowadays I do just feel okay. So it has helped me.”* (23 years old, never married, secondary school not completed- Month 6 IDI.)

### Themes table

Table 2 summarizes the occurrence of barriers and facilitators to doxyPEP adherence across different time points (months 1, 6, and 12) and methods (IDIs and FGDs), highlighting common and unique themes at each stage.

**Table 2:** Barriers and Facilitators Identified in The Interviews, Grouped by Time Points

Theme	Month 1 (Initial IDIs)	Month 6 (IDIs)	Month 12 (IDIs at Exit)	FGDs (after study exit)
**Barriers**				
Side Effects	✓ (28/40)	✓ (15/40)	✓ (25/40)	✓
Stigma and Privacy Concerns	✓ (21/40)	✓ (27/40)	✓ (15/40)	✓
Forgetfulness and Routine Disruptions	✓ (8/40)	✓ (16/40)	✓ (20/40)	✓
Partner Influence and Reactions	✓ (13/40)	✓ (14/40)		✓
Pregnancy and Health Concerns		✓ (5/40)		
Access Issues			✓ (5/40)	
**Facilitators**				
Perceived Effectiveness/Protection	✓ (12/40)	✓ (15/40)	✓ (30/40)	✓
Use of Discreet Pill Carriers	✓ (10/40)	✓ (16/40)	✓ (15/40)	✓
Social Support and Encouragement	✓ (9/40)	✓ (14/40)	✓ (20/40)	✓
Routine Integration	✓ (7/40)	✓ (14/40)	✓ (25/40)	✓
Health Education and Provider Support		✓ (8/40)		
Fear Reduction		✓ (1/40)		
Positive Experiences and Benefits		✓ (7/40)		

## Discussion

The experiences of young women participating in the dPEP Kenya Study offer valuable insights into the factors that hinder and facilitate adherence to doxyPEP, contributing to the general understanding of the preventive measures for STIs. The first randomized trial of doxyPEP among cisgender women did not show efficacy, in contrast to high efficacy among people who were assigned male sex at birth, and objective drug testing indicated that many participants were not taking doxyPEP. The success of biomedical interventions, like doxyPEP, will only be effective if we can address both personal and contextual factors to facilitate adherence.

Side effects were highlighted as one of the major barriers to adherence. This finding is consistent with other PrEP studies, where side effects have been noted as a barrier to adherence and a contributing factor to drug discontinuation [29]. The pill burden of additional medicines, especially in combination with HIV PrEP, was another common obstacle in adhering to the treatment. Participants expressed that keeping a multi-pill regimen was challenging, which, in turn, negatively affected their commitment to doxyPEP. The same issue also influences adherence in other populations as pill burden reduces adherence [30, 31]. Addressing the pill burden includes the development and use of longer-acting PrEP formulations such as injectable PrEP or multipurpose prevention technologies that combine HIV prevention with treatment for other conditions, reducing the overall number of medications taken simultaneously [32, 33].

The stigma surrounding doxyPEP use, particularly concerns about being judged for taking medication associated with STI treatment, emerged as a significant barrier. Participants worried about how others, including their sexual partners, might perceive their use of doxyPEP leading some women to avoid the medication altogether. Stigma related to sexual health interventions is well-documented, especially among women in sub-Saharan Africa, and continues to hinder prevention efforts [34, 35]. Strategies to reduce stigma and enhance education about doxyPEP’s role in STI prevention are critical to overcoming these barriers.

The time-specific patterns in barriers illustrate the changing participant experiences with doxyPEP throughout the study. For instance, some issues like side effects and stigma remained constant, while others like pregnancy-related health concerns and access difficulties dominated at months 6 and 12 respectively. For example, pregnancy concerns surfaced more often at month 6 as participants became aware of possible dangers to the fetus, while access issues were more prevalent in month 12 due to greater travel or transportation difficulties. These synchronized patterns highlight the need for ongoing, customized support—especially regarding proactive safety instructions during pregnancy and maintaining accessibility despite changes in routine or location—throughout the duration of doxyPEP use.

A number of factors facilitating doxyPEP adherence were also identified, including optimism about doxyPEP effectiveness, social support, and the use of discrete pill carriers. The participants who held the view that doxyPEP was useful in STI prevention were high adherers compared to those who held contrary views, a finding consistent with the literature [36]. Support from family and friends also facilitated adherence, demonstrating how support from social networks is critical in promoting healthy behavior. Participants who disclosed their use of doxyPEP often received emotional encouragement and practical support from friends, family and partners. This appeared to positively influence their consistency with the intervention and highlights the value of supportive social networks in facilitating adherence to biomedical HIV and STI prevention tools. Prior studies have emphasized the importance of social support as a facilitator of health intervention adherence especially in resource-poor settings [37].

Small, empty cosmetic containers are a cost-effective intervention that could be easily employed to promote adherence when used as discreet pill carriers. These pill carriers also emerged as important facilitators in the sense that they enabled participants to take their medication while ensuring that limited attention was drawn to the act. This finding demonstrates the need for privacy in enhancing adherence, especially where stigma serves as a barrier to sexual health interventions. Similarly, other studies on adherence to PrEP have shown that enabling users to keep their PrEP use discreet helps users adhere to the regimen [38].

### Implications for Future Research, Practice, and Policy

The results of this study have several implications for future research, clinical practice, and public health policy. Future research should continue to explore barriers specific to cisgender women in relation to other biomedical prevention methods, such as PrEP. Interventions aimed at addressing these barriers—including management of side effects, stigma reduction through education campaigns, and strategies to lessen pill burden—warrant further investigation.

There is a need for healthcare providers to prioritize patient-centered conversations with individuals regarding potential barriers to doxyPEP adherence. This helps consider that each patient is different and has unique needs, which will allow for management of side effects to be tailored effectively. Integrating doxyPEP into the current sexual and reproductive services can enhance its acceptability and reduce associated stigma.

Additionally, policymakers should be encouraged to formulate and put into action strategies that would assist in the integration of doxyPEP into public health programs. These strategies include the provision of financial support for education campaigns, the provision of subsidized services to enhance accessibility, and the creation of appropriate clinical guidance for the implementation of the intervention. All this should be preceded by education, which will cover the advantages, the expected barriers, and practical aspects so that both patients and practitioners are equipped to make informed choices.

### Limitations

A key limitation of this study is that it primarily relied on participants’ self-reported experiences, which may introduce social desirability bias, especially when discussing sensitive topics such as sexual partnerships. This could lead participants to provide responses they believe are more socially acceptable. Our ongoing presence in the community and the trust developed enabled us to obtain findings that were meaningful and reflected women’s realities. For example, women were open about having multiple relationships and so they did share sensitive information that has important implications for future health interventions and healthcare policy.

Additionally, the study did not account for food insecurity during the peak of the COVID-19 pandemic, which may have influenced participants’ experiences in relation to the side effects of medications. Without assessing this factor, it is difficult to determine whether COVID-19 lockdowns also contributed to some participants taking doxyPEP on an empty stomach.

## Conclusion

The full benefit of biomedical prevention tools, including doxyPEP, can only be fully realized when barriers to uptake and adherence are properly addressed. For this study, primary challenges included anticipated social stigma, forgetfulness, fear of partner reactions and side effects. These barriers may help explain the null results of the trial, as inconsistent use could have limited the intervention’s effectiveness. Addressing these barriers by building on the identified facilitators through community-driven support, clear communication about side effect management, and simplified dosing strategies is essential. While doxyPEP is a promising self-directed intervention, its success is still shaped by broader social and structural factors that must be considered in future implementation efforts.

## Clinical Trial

This trial was registered in the ClinicalTrials.gov under registration number, NCT04050540 on 06th August 2019.

## Supplementary Information

Supplementary Information 1.

Supplementary Information 2.

Supplementary Information 3.

## Data Availability

De-identified transcripts generated and/or analysed during the current study are available from Dr. Jenell Stewart (jenell.stewart@hcmed.org) upon reasonable request.

## Abbreviations

DoxyPEP/dPEP: Doxycycline Post-Exposure Prophylaxis
dPEP Kenya Trial: Doxycycline Post-Exposure Prophylaxis Kenya Trial
IDI: In-depth Interview
FGD: Focus Group Discussions
HIV: Human Immunodeficiency Virus
PrEP: Pre-Exposure Prophylaxis
STI: Sexually Transmitted Infections
USA: United States or America
MSM: Men who have Sex with Men
