# Supplementary material for: Understanding barriers and facilitators to doxycycline post-exposure prophylaxis adherence among young women in western kenya: a qualitative study
Source: BMC Infect Dis. 2025 Jul 1;25:855. doi: 10.1186/s12879-025-11209-6 (PMC12220084; doi:10.1186/s12879-025-11209-6)
Supplement: Supplementary file 1 — Supplementary Information 1. [file 12879_2025_11209_MOESM1_ESM.docx]

**Appendix A. Members of the dPEP Kenya Trial Team**

**Kenya Medical Research Institute, Kisumu, Kenya**
Alfred Odira¹, Perez Ochwal¹, Lydia Adiema¹, Marion Hewa¹, Elizabeth Koyo Akumu¹, Linda Aswani¹, Lawrence Juma¹, Violet Kwach¹, Felix Mogaka¹, Vincent Momanyi¹, Alfred Obiero¹, Loice Okumu¹, Victor Omollo¹, Christine Otieno¹, Greshon Rota¹

**University of Washington, Seattle, USA**
Jacqueline M. Amira^3^, Justice Quame-Amaglo^3^, Ruanne Barnabas^3^, Jennifer Baugh^3^, Jade Boyer^3^, Connie Celum^3^, Kristin Cicciarella^3^, Deborah Donnell^3^, Daphne Hamilton^3^, Harald Haugen^3^, Rachel E. Johnson^3^, Toni M. Maddox^3^, R. Scott McClelland^3^, Susan A. Morrison^3^, Colin S. Pappajohn^3^, Elena Rechkina^3^, Caitlin Scoville^3^, Tina Sesay^3^, Olusegun O. Soge^3^, Kathy Thomas^3^, Jane Simoni^3^, Vianey Vazquez Venegas^3^

**Affiliations**:
^1^Kenya Medical Research Institute, Centre for Microbiology Research, Kisumu, Kenya
^3^University of Washington, Departments of Global Health, Seattle, USA
