# Supplementary material for: Understanding barriers and facilitators to doxycycline post-exposure prophylaxis adherence among young women in western kenya: a qualitative study
Source: BMC Infect Dis. 2025 Jul 1;25:855. doi: 10.1186/s12879-025-11209-6 (PMC12220084; doi:10.1186/s12879-025-11209-6)
Supplement: Supplementary file 2 — Supplementary Information 2. [file 12879_2025_11209_MOESM2_ESM.docx]

**dPEP FGD guide**

Thank you for finding time to come for this group discussion that we hope to engage you on your experiences and opinions on taking doxycycline postexposure prophylaxis (dPEP). Feel free to share what you know or have heard about other people’s experiences during their participation in the study.

1. Tell me about your opinion of using doxycycline as PEP to prevent STIs?
   1. What do you like about it? Can you think of an example of when dPEP was helpful?
   2. What do you dislike about it? Can you think of an example of when dPEP was harmful?
2. Most people have challenges taking medication, how were the challenges with taking dPEP different from taking HIV PrEP?
   - 1. What did you think of morning-after dosing (of dPEP) compared with daily dosing (of PrEP)?
     2. How was the decision to take dPEP different from when to take PrEP?
   1. How did you decide when to take dPEP?
      1. Probe on different dPEP usage after different partners? Why?
      2. Probe on how taking dPEP made people feel (both physically and mentally)
3. As you may know the purpose of dPEP study was to find out if taking dPEP medicine can protect people from getting an STI, do you have any general comments on why you think that we did not we find the medicine to be effective at preventing STIs? Kindly share with us.
