# Supplementary material for: Understanding barriers and facilitators to doxycycline post-exposure prophylaxis adherence among young women in western kenya: a qualitative study
Source: BMC Infect Dis. 2025 Jul 1;25:855. doi: 10.1186/s12879-025-11209-6 (PMC12220084; doi:10.1186/s12879-025-11209-6)
Supplement: Supplementary file 3 — Supplementary Information 3. [file 12879_2025_11209_MOESM3_ESM.docx]

| **Purpose** | There are three main goals of the serial in-depth interviews:   - To understand acceptability of and adherence to doxycycline post-exposure prophylaxis (dPEP) for STI prevention - To understand gendered power dynamics in primary relationships and how that relates to adherence - To understand sexual behavior patterns among women taking dPEP for STI prevention | | |
| --- | --- | --- | --- |
| **Instructions** | For the interviewer to understand; how to read this IDI Discussion Guide  There are 2 levels of questions:  **Initial topic questions:** These questions will be numbered. They address the topics that you as the interviewer must ask and discuss with participants. The questions are suggestions for getting the discussion going. You are not required to read them exactly as they are written, but they are written to ensure some consistency across IDIs. You may adapt the questions and/or ask them in a different order, depending on how the interview develops. However, you will have to ensure that by the end of the interview, all the topics and key themes have been covered. A local-language translation of these questions should be provided on this document in the space provided.  **Probes:** They are indicated with a bullet. The interviewer should ensure that key topics listed in the guide have been addressed/discussed during the interview; however, the probes are meant to serve as suggestions for the interviewer to draw from rather than a strict list of questions that must be asked. So, depending on what has already been discussed, and the IDI context, you may ask these probes or not or may phrase probes differently to try and better understand what the participant is trying to communicate.  **Note:** Instructions/suggestions to interviewer are in *italics*.  [*Warmly*.] Good morning/afternoon. My name is _________________. Thank you so much for your willingness to be a part of this discussion. You are one of a small group of women that we have invited to work with us understand your experiences with dPEP. By helping us to understand you and your world, you can help us design outreach and services for STI prevention that can help many other women in Kisumu, Kenya, and other countries.  For today, I am interested in learning more about what you think about dPEP and some information about your relationships and sex partners. As you read in the consent form, everything you say in this interview is confidential. That means your words are not linked to your name.  As we move forward, know that all of your thoughts are important to us. You do not need to answer all the questions and please feel free to skip any question.  Do you have any questions before we begin?  To help me to take notes later, I’d like to record our conversation *[confirm participant consented to audio recording]*. | | |
| *Theme* | **#** | **Main question** | **Probes (as needed)** |
| *To start our discussion today, I would like to know more about you.* | | | |
| **Personal questions** | 1 | What is the primary language that you speak? | - Are there other languages that you feel comfortable communicating in? |
|  | 2 | Do you have a preference about being interviewed by a man or woman? | - Do you feel more comfortable talking about your personal life with people of different genders? |
|  | 3 | How did you hear about this study? |  |
|  | 4 | What is the study you’re participating in about? | - How do the dPEP pills work? What do they do? |
| *As part of your participation in this research study, you were given dPEP to take after condomless sex. I would like to hear more about your thoughts on taking dPEP* | | | |
| **Experiences with dPEP** | 5 | Generally, what are your thoughts or feelings about taking dPEP? |  |
|  | 6 | Have you been able to take any of your dPEP pills? Please tell me about that. | - Where do you keep your pills? - Usually, when do you take dPEP? - Do you take it as soon as you finish sex or later? |
|  | 7 | How easy or hard do you think is it for you to use these dPEP medicines? | - What makes it easy/hard to remember doses? - How important is it for you to take the pills in private? - Normally, what do you do with the pills when you traveling? - Do you use a pill carrier? |
|  | 8 | We know it can sometimes be hard to remember to take dPEP as directed. Tell me about a recent time that you struggled to be able to take your pills. | - How well do you think other women in this study did with taking dPEP? |
|  | 9 | How do you feel about taking dPEP? | - Anything about taking dPEP that you dislike? |
|  | 10 | How well do you think dPEP medication helps you prevent infections that would have bothered you? | - How confident are you that you can use these dPEP medicines to prevent you from getting infected with STIs? |
|  | 11 | How do you feel about taking PrEP for HIV prevention and dPEP for STI prevention? | - Does taking PrEP help you remember when to take dPEP? Or vice versa? |
|  | 12 | Did you tell anyone that you are taking dPEP? | - To whom did you talk about dPEP? - What was their reaction? - How did you feel? Did you feel stigmatized or empowered? - Did it change the way you decided to take your dPEP? - OR - Share with the reason you did not want anybody to know that you were taking dPEP. |
|  | 13 | Has anyone you didn’t mean to find out about your dPEP learned about you taking dPEP? For example, someone saw your pills. | - How did this happen? - What was their reaction? - Did it change the way you decided to take your dPEP? - OR - How have you been able to take dPEP for this long without disclosing use of dPEP? |
|  | 14 | Have you ever shared your dPEP pills with anyone? Tell me more about that. | - Who did you share your dPEP with? - Why did you share your dPEP pills? |
|  | 15 | After this study if dPEP were available from your regular PrEP prescriber, would you want to continue dPEP? | - Why or why not? |
|  | 16 | Do you think your friends would want to take dPEP? | - Why or why not? |
| *In a relationship or marriage, many decisions need to be made. I’d now like to ask you some questions about the way in which decisions are made in your primary relationship, and the roles you and your partner/husband play in making those decisions. May I proceed?* | | | |
| **Decision-Making & Power** | 17 | Tell me about your sexual relationships | - Do you have a primary partner (husband or boyfriend)? - Have you ever had more than one sexual partner at any given time? Tell me more about that |
|  | 18 | Share with me how you feel about making decisions in your relationship? | - Do you have opportunities to make decisions in your relationships? - Who makes more decisions in your relationship? You? Your partner? Both? - Why? |
|  | 19 | Tell me incidences where you discuss STDs or HIV in your relationship | - Who starts the conversation? - If you were to refuse sex without a condom, how do you think your partner/husband would respond? |
|  | 20 | Who influences how or if you take dPEP? | - Your partner? Parents? Friends? - Is it the same or different for PrEP? |
|  | 21 | In what ways does your partner control the medicines you take? | - Do you feel that you could take dPEP even if your partner did not want you to? - If you had more power in your relationship would it be easier to take dPEP? |
| *Some people have noticed changes in their sex life after starting PrEP, and I’d like to ask about the impact of dPEP on your sex life.* | | | |
| **Impact on sexual behavior** | 22 | Young women have reported having different kinds of partners (boyfriends, girlfriends, main, casual, paying (both being paid for and paying for sex). Tell me, what types of partners do you have? | - Did that change after starting dPEP? |
|  | 23 | How has dPEP affected your sex life? | - If not, why? - Has dPEP changed your stress and fear about getting an infection from sex? - Are you having more or less sex because of dPEP? |
|  | 24 | What do you think about being asked about sex or “dancing” via text message each week? | - Is it more or less comfortable reporting how many days you have sex each week via text? |
|  | 25 | How hard was it to report accurately via text message? |  |
| **Conclusion** | 26 | Which questions were too hard, unclear, or unpleasant to answer? | - - How was it to participate in the interview? |
|  | 27 | Were there any other issues related to these topics that you think we should know about or that you wanted to talk about? |  |
| *Those are all the questions that I had. Thank you. [Turn off recorder]: Provide participant with [INSERT PAYMENT AMOUNT] for participation and ask for signature. If you have any further thoughts that you think may be helpful to the project, or if you know anyone who would be interested in participating in this research please do not hesitate to contact us.* *We’re looking forward to seeing you at your second interview in a few months’ time. Thank you again. It is a pleasure to work with you on this project.* | | | |
